# β-Lactam Dosing in Critical Patients: A Narrative Review of Optimal Efficacy and the Prevention of Resistance and Toxicity

**DOI:** 10.3390/antibiotics11121839

**Published:** 2022-12-18

**Authors:** João Gonçalves Pereira, Joana Fernandes, Ana Rita Duarte, Susana Mendes Fernandes

**Affiliations:** 1 Hospital Vila Franca de Xira, 2600-009 Vila Franca de Xira, Portugal; 2 Grupo de Investigação e Desenvolvimento em Infeção e Sépsis, 4450-681 Matosinhos, Portugal; 3 Centro Hospitalar de Trás-os-Montes e Alto Douro, 5000-508 Vila Real, Portugal; 4 Nova Medical School, Universidade NOVA de Lisboa, 1099-085 Lisbon, Portugal; 5 Clínica Universitária de Medicina Intensiva, Faculdade de Medicina, Universidade de Lisboa, 1649-004 Lisboa, Portugal

**Keywords:** β-lactam, pharmacokinetics, pharmacodynamics, critical care, dosing, organ failure, toxicity, resistance

## Abstract

Antimicrobial prescription in critically ill patients represents a complex challenge due to the difficult balance between infection treatment and toxicity prevention. Underexposure to antibiotics and therapeutic failure or, conversely, drug overexposure and toxicity may both contribute to a worse prognosis. Moreover, changes in organ perfusion and dysfunction often lead to unpredictable pharmacokinetics. In critically ill patients, interindividual and intraindividual real-time β-lactam antibiotic dose adjustments according to the patient’s condition are critical. The continuous infusion of β-lactams and the therapeutic monitoring of their concentration have both been proposed to improve their efficacy, but strong data to support their use are still lacking. The knowledge of the pharmacokinetic/pharmacodynamic targets is poor and is mostly based on observational data. In patients with renal or hepatic failure, selecting the right dose is even more tricky due to changes in drug clearance, distribution, and the use of extracorporeal circuits. Intermittent usage may further increase the dosing conundrum. Recent data have emerged linking overexposure to β-lactams to central nervous system toxicity, mitochondrial recovery delay, and microbiome changes. In addition, it is well recognized that β-lactam exposure facilitates resistance selection and that correct dosing can help to overcome it. In this review, we discuss recent data regarding real-time β-lactam antibiotic dose adjustment, options in special populations, and the impacts on mitochondria and the microbiome.

## 1. Introduction

Treatment prescription in critically ill patients represents a complex challenge, as drug dosing might be a game changer for the progression towards organ failure or death. On the other hand, changes in organ perfusion and function lead to unpredictable pharmacokinetics and side effects. In the end, adequate dosing in critically ill patients may be especially difficult to achieve [1].

Antibiotics are among the most used drugs in critically ill patients, and β-lactams are considered the most useful class among them [2]. The overall consumption of antibiotics shows a wide variation according to region, ranging from 4.4 to 64.4 defined daily doses per 1000 inhabitants. The antibiotics in the World Health Organization’s watch category may account for more than 50% in some countries [3]. Moreover, this trend may be increasing [4], which raises the problem of overuse and emerging resistance.

Achieving the rapid killing of bacteria responsible for infections is of the utmost importance to resolving infection and sepsis [5]. However, changes in antibiotic pharmacokinetics may easily lead to either underexposure, with therapeutic failure and the selection of resistant mutants [6], or overexposure, with significant toxicity risk [7], which is often unrecognized in a multiorgan failure patient [8].

Accordingly, the doses of antibiotics can no longer be seen as fixed from the beginning to the end of treatment, and real-time adjustments to the patient’s condition are critical. The continuous infusion of β-lactams [9] and the therapeutic monitoring of their concentration [10] have both been proposed to improve efficacy, but strong data to support their use are still lacking. The knowledge of the pharmacokinetic/pharmacodynamic (PK/PD) targets is poor and is mostly based on observational data [11], which may make clinical decisions difficult. Conflicting data suggest that lower PK/PD targets could suffice to improve patients’ prognoses [12].

In patients with renal or hepatic failure, adequate dosing of antibiotics can be even more challenging because not only is clearance decreased but adaptative changes may also influence drug concentration and distribution [13]. Extracorporeal circuits, often used in this population, are well known to influence drug concentration, either sequestering the drug in the circuit or eliminating it from the body [14]. Their intermittent usage may further increase the dosing conundrum.

Increasing the dose to achieve adequate bacteria killing is tempting, especially in septic shock patients. However, recent data have emerged linking overexposure to β-lactams to undesired effects, especially targeting the central nervous system [15], mitochondria [16], and the microbiome [17]. These can lead to a delay in patient recovery or even lead to a worse prognosis. Data suggesting increased mortality in patients with repeated antibiotic exposure [18] are of special concern. In addition, it is well recognized that β-lactam exposure facilitates resistance selection, and correct dosing may help to overcome it [19].

Most reviews on this subject have focused on using high doses to achieve increased bacterial clearance. However, concerns about overexposure and toxicity should also be addressed.

In this narrative review, we aim to discuss the challenges of β-lactam dosing, focusing not only on efficacy but also on the prevention of toxicity and the emergence of resistance. The need to address intra- as well as interpatient PK variability, namely promoting personalized dosing, is also discussed.

## 2. Dose of β-Lactams in Critical Illness

### 2.1. Pharmacokinetics of Sepsis

#### 2.1.1. Changes in Antibiotic Pharmacokinetics in Critically Ill Patients

Critically ill patients commonly present significant pathophysiological changes that modify the PK of antimicrobials [20]. Particularly in septic shock, the blood flow of the gastrointestinal tract and subcutaneous tissue may be severely reduced and shunted to vital organs [21] such as the brain and heart. This compromises reliable drug absorption through these routes. As a result, the intravenous administration of antimicrobials is commonly recommended [22].

These changes, noted in sepsis, result from a significant fluid shift from the intravascular compartment to the interstitial space [23]. These shifts are the product of inflammatory endothelial damage and increased capillary leakage [24], which can be aggravated by aggressive intravenous volume replacement. Hypotension and hypoperfusion can also damage organs and tissues, further changing the antibiotic concentration and PK.

The antibiotics’ volume of distribution (Vd) is often increased [21,25], and consequently, as β-lactams are hydrophilic antimicrobials, there is a need for higher loading doses to achieve the same adequate therapeutic concentrations.

The β-lactams’ Vd is also influenced by protein-binding modifications [26], particularly albumin, one of the main plasma-binding proteins for many drugs [27]. Septic patients commonly have decreased concentrations of albumin, which directly impacts the PK of antibiotics. Hypoalbuminemia increases the unbound antibiotic concentration [26], which leads to a lower, probably suboptimal, antimicrobial concentration. These should be accounted for if therapeutic drug monitoring (TDM) is used, and the direct measurement of free drug levels should be preferred [28,29].

Not only do these pathophysiological changes directly influence antibiotic concentrations, but therapeutic interventions can also cause dramatic modifications of the antibiotic PK [30]. Invasive mechanical ventilation, abdominal surgery, and high-dose vasopressors are among the interventions that contribute to fluid shifts and changes in drug concentration.

Acute kidney injury and a reduced glomerular filtration rate [28] are also common complications of sepsis. Accordingly, decreased drug clearance (and a longer half-life) may occur, making antibiotic PK and concentrations largely unpredictable [31]. In addition, the evaluation of renal clearance may be quite challenging due to frequent changes in the fluid balance and the poor predictive value of serum creatinine [31].

**Figure 1:**
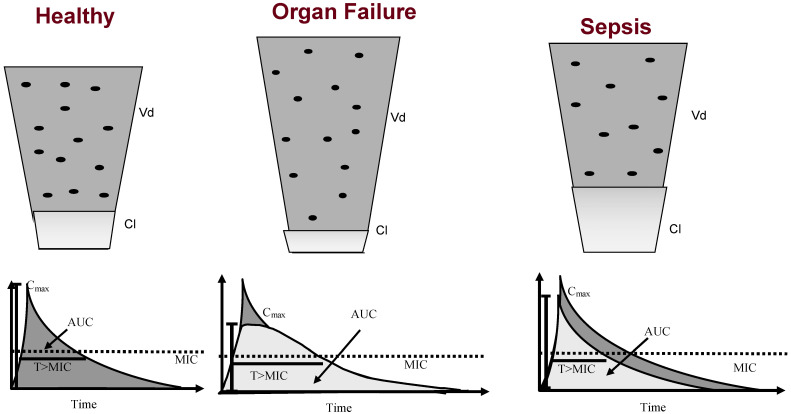
Changes in pharmacokinetics of drugs (including antibiotics) in critically ill patients. During organ failure, changes in water metabolism often lead to an increase in the volume of distribution and a lower concentration. Conversely, clearance is commonly decreased, and a longer half-life is expected. In sepsis, both the volume of distribution and clearance may be increased, and all pharmacokinetic/pharmacodynamic targets are decreased. Vd—Volume of distribution; Cl—Clearance; Cmax—Maximum peak concentration; AUC—Area under the curve; T > MIC—Time above minimal inhibitory concentration (adapted from [25]).

Even in patients without acute kidney injury, the antibiotics’ serum concentrations may be severally altered due to augmented renal clearance [20,31]. This is the case of antibiotics predominantly excreted by the kidney, such as β-lactams (Figure 1). During augmented renal clearance, β-lactam elimination is increased, and the consequent subtherapeutic concentrations can jeopardize the efficacy of antibiotic therapy [32].

These PK changes are dynamic, and daily changes are very common, leading to the frequent need to adapt the antibiotic dosage. Antibiotic concentrations can be quite unpredictable, and failure or toxicity, which are directly related to a change in the concentration, may become frequent [21].

Furthermore, the relationship between the amount of drug being administered and the time of perfusion, either by conventional intermittent dosing or extended or continuous infusion, may influence its PK.

#### 2.1.2. β-Lactam Antibiotic Mechanisms of Action

β-lactam antibiotics act through a pathway that blocks the growth and replication of bacteria, leading to their death. Their main mechanism of action is the inactivation of enzymes located in the bacterial cell membrane, known as penicillin-binding proteins, which are involved in cell wall synthesis [33]. It is well known that different β-lactam antibiotics may preferentially bind to and inhibit specific penicillin-binding proteins according to their spatial structures [34], which leads to different efficiencies in the killing of microorganism. These antibiotics have bactericidal properties against replicating bacteria, meaning they kill the microorganisms through the inhibition of bacterial cell wall synthesis, which is necessary for replication, leading to their lysis after division is complete [33].

Although all β-lactams share the same β-lactam ring, they present different structures and bacteria-sensitive profiles, leading to their classification in different classes, namely penicillins, cephalosporins, monobactams, and carbapenems [35,36]. These changes in antibiotic structure are directly related to their stability against bacterial resistance mechanisms (especially β-lactamase enzymes).

These antibiotics are time-dependent, that is, their activity is mainly related to the time their concentration is above the minimal inhibitory concentration (MIC). Different thresholds have been determined for the diverse classes of β-lactams in vitro or in animal models [37]. Bacteria killing is ensured as soon as this threshold is attained but does not increase significantly with higher concentrations [38]. Moreover, no significant post-antibiotic effect is generally noted [38,39].

#### 2.1.3. Dose Modulation

The concept of dose modulation consists of a broader and more innovative view of de-escalation. This includes the front-line variability in the antibiotic dosage, according to patient and microorganism characteristics, followed by its reduction after clinical response and patient recovery. This means concentrating the largest weight of antibiotics at the front end [40], when the microbial load is higher and the PK changes pose the highest risk of underdosing, and reducing the antibiotic dose when the sepsis syndrome is improving [22], guided by PK and PD data.

In fact, in patients responding to therapy and recovering from infection, there is commonly a hemodynamic improvement, with the weaning of vasopressors, a negative fluid balance, and the normalization of cardiac, renal, and hepatic functions, which also lead to progressive antibiotic PK normalization [28].

Therefore, after hemodynamic recovery, if the patient is improving and there is evidence of a clinical response, it is probably safe to reduce the antibiotic dose to reduce the risk of antibiotic toxicity. Furthermore, there is a large amount of evidence that the duration of antibiotic treatment can be largely decreased [41,42], which may help to control the overall antibiotic exposure and the emergence of resistance.

This strategy, along with the use of a broad-spectrum combination antibiotic therapy, may help to overcome the limitations to its efficacy posed by the large inoculum [43], the PK changes, and the decreased antibiotic susceptibility [44].

#### 2.1.4. Therapeutic Drug Monitoring

Conventional antibiotic dosing, accounting only for changes in renal or hepatic clearance, may be prone to failure in complex critically ill patients [24].

In fact, despite the PK changes, the dose is usually maintained throughout the treatment course. With an expanding knowledge of the relationships between antimicrobial dosing, pharmacokinetic/pharmacodynamic (PK/PD) exposure, and patient outcomes [22], there is now a strong rationale to individualize antimicrobial dosing in critically ill patients with the aid of TDM [10].

As already pointed out, antibiotic dosing in critically ill patients is especially challenging due to the increased Vd [21] and the rapid changes in renal and hepatic clearance. Moreover, bacterial inoculum, commonly much higher in critically ill patients, may also influence the bacteria-killing kinetics and may contribute to therapeutic failure [28]. Consequently, dose individualization according to the PK of the antimicrobial and the patient’s unique clinical characteristics may have a strong impact on achieving the optimal therapeutic exposure based on the bacterial susceptibility. Theoretically, this may help to maximize the killing of bacteria, minimize toxicity, and prevent the emergence of resistance, which is especially important when dealing with patients at a high risk of death and in environments with escalating antibiotic resistance [40].

Therefore, TDM for β-lactams has been suggested for routine implementation in the treatment of critical illness. Accordingly, TDM would help to implement the dose modulation strategy, facilitating the early attainment of the maximal tolerable dose [45] of the selected antibiotic. This would facilitate avoiding initial antibiotic underdosing on one hand, potentially improving the antibiotic effectiveness and, on the other hand, promoting biosafety, decreasing the antibiotic exposure time, and avoiding overexposure and toxicity [40].

The most conventional and practical method to use TDM is based on the evaluation of a single PK sampling at the end of a dosing interval (a trough sample with a minimal concentration). The measured serum concentration is compared against a therapeutic target range, and dosage adjustments are performed. This constitutes the easiest methodology, but it is also the least accurate for dosing adjustments. Limited sampling strategies incorporate up to three samples, providing more informative concentration time points for PK description [46,47]. These can be conjugated with dosing nomograms, which are ideally tested on similar populations [48], and can incorporate data on organ function, along with the PK/PD data, providing a more accurate method for dosing adjustment [21]. The dosing of the total concentration of the drug may be misleading and the measurement of the free drug concentration should be preferred [26].

Given the safety profile of β-lactams, TDM has been proposed to focus on maximizing efficacy through the achievement of adequate therapeutic exposures. As observational data suggested larger-than-conventional PK/PD targets, several TDM studies focused on achieving 100% values for the time with a free drug concentration above the MIC (100% *f*T > MIC) or even more than four times above that target [12,49]. The measurement of bacterial MIC values, drug sampling, and the use of nomograms may help to achieve these predefined therapeutic targets [29,50].

In a large study, assessing the TDM of β-lactams in 330 critically ill patients, Wong et al. were able to achieve these more ambitious therapeutic targets in 63.4% of patients [51]. However, in the population with the microbiological documentation of an infection, there were no differences in outcomes (odds ratio: 0.88 (95% CI: 0.40–1.91); *p* = 0.74 and odds ratio: 0.67 (95% CI: 0.29–1.55); *p* = 0.35 for 100% *f*T > MIC and 100% *f*T > 4 × MIC, respectively) [51].

The potential benefits of TDM are mainly related to the existence of a recognized relationship between a serum drug concentration and the intended effect. Moreover, this clinical benefit is mostly based on the possibility of having a rapid and inexpensive method to measure drug concentrations reliably and with a short turnaround time (allowing real-time dosing adjustment). As of today, this may not be the case for β-lactam antibiotics.

Dynamic changes in therapeutic targets according to the mode of antibiotic administration (either continuous infusion or conventional intermittent dosing) have been unveiled in an in vitro model [52]; furthermore, excessive reliance on MIC determination [53] may lead to false assumptions, which may explain the incongruencies between achieving PK/PD targets but attaining inadequate clinical outcomes.

#### 2.1.5. Continuous Infusion of β-Lactams

There is increasing evidence that front-line antibiotic inappropriateness is common and may have a significant impact on the outcomes of patients with severe infections and septic shock [54]. An appropriate spectrum of antibiotic therapy may be insufficient if adequate exposure is missed [55]. However, the PK changes that occur in infected critically ill patients often lead to significantly different antibiotic exposures, which have an impact on their effectiveness.

In clinical practice, the major benefit of antibiotics, rapid bacteria killing, seems to be mostly concentrated to the first days of therapy. For β-lactams, the early achievement of 100% *f*T > MIC with continuous or extended infusions [56] during the first hours of therapy is probably safe and may improve clinical outcomes. Increasing the time of β-lactam perfusion is associated with extended *f*T > MIC [57], although the total exposure remains the same.

**Figure 2:**
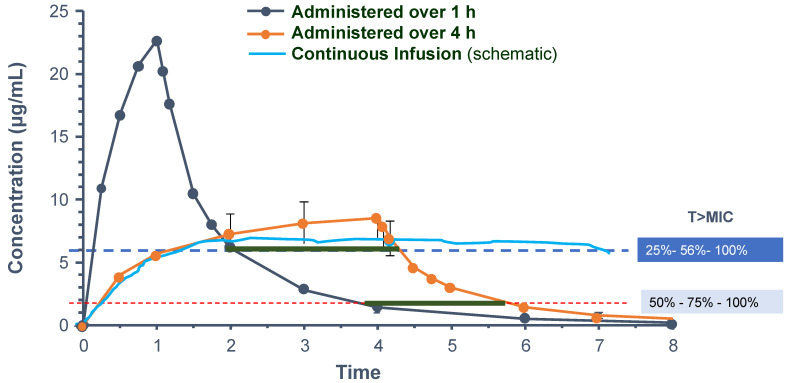
Concentration–time curves of β-lactams according to administration times of 1 or 4 h and a schematic representation of continuous infusion. Conventional 1 h bolus dosing achieved high peak concentrations, while prolonged or continuous infusions had more stable, albeit lower, concentrations. However, these also had increased time above the minimal inhibitory concentration and faster killing kinetics. This could be especially important when bacteria are less sensitive to the antibiotic (adapted from [57]).

In critically ill patients, higher targets have been proposed, especially for β-lactams, including 100% of the time over a concentration as high as 4 × MIC [58]. Consequently, extended or even continuous infusions of these antibiotics have been proposed to be able to achieve these higher targets and improve clinical outcomes (Figure 2). However, theoretically, the plateau concentration of β-lactams, which is associated with continuous infusion, may be below the efficacy threshold 100% of the time, and this could lead to the worst outcome.

The strategy of the extended infusion of β-lactams was used in a study addressing piperacillin/tazobactam in patients infected with Pseudomonas aeruginosa. In this before–after study, a significant decrease in mortality in the most severe group of patients was noted (12.2% vs. 31.6%, respectively; *p* = 0.04) [59]. However, this very outcome was not found in other studies [60,61,62] or large meta-analyses [63,64], reinforcing the complexity of the PK/PD targets.

A meta-analysis of the individual data of three prospective studies comparing continuous infusion with intermittent doses of β-lactams [65] unveiled a small but significant benefit of continuous infusion in reducing in-hospital mortality (19.6% vs. 26.3%, relative risk: 0.74 (95% confidence interval: 0.56–1.00), *p* = 0.045). Some answers may come from a large multicenter trial with a similar protocol that is just finishing recruitment [66].

It is important to note that the limited data relating to PK/PD targets and outcomes should not be viewed as proof of a lack of benefit. While the attainment of any antibiotic concentration target does not guarantee an improved outcome per se, using individually guided dosing optimizes the probability of achieving adequate bacterial killing [67]. Variability in targets (such as the organisms’ MIC values), intra- and interindividual variability in PK, β-lactam “silent” toxicity, and possible differences in the adequate PK/PD targets according to the bacteria and type of infection point to the complexity of this process.

Furthermore, the measurement of the clinical response is also not standardized. Decreases in biomarkers [68,69] or even in the bacterial inoculum [70] may be used, but their impacts on antibiotic management are still unclear.

### 2.2. Antibiotic Pharmacokinetics in Organ Failure and Extracorporeal Support

**Figure 3:**
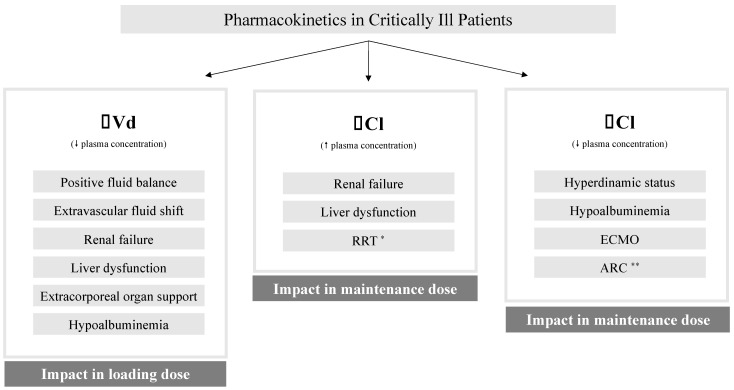
Main pharmacokinetic changes of β-lactam antibiotics in critically ill patients. * Especially common in drugs with high volumes of distribution (>1 L/Kg) or high protein binding (>80%); ** More common in young males with trauma, sepsis, burns, hematological malignancy, or pancreatitis. Vd—volume of distribution; Cl—clearance; RRT—renal replacement therapy; ECMO—extracorporeal membrane oxygenation; ARC—augmented renal clearance (creatinine Cl > 130 mL/min); ↑—Increased; ↓—Decreased. Adapted from [28].

In septic patients with organ failure, it is also challenging to ensure therapeutic and nontoxic antibiotic exposures (Figure 3) [49]. To guarantee adequate therapeutic levels and ensure optimal bacterial killing, higher-than-standard doses should also be considered, namely a loading dose in the initial phase of sepsis [13]. Afterward, subsequent dosing must be guided by drug clearance, mainly for renal and hepatic dysfunction [13]. Extracorporeal circulation devices also influence antibiotic PK, increasing the Vd (due to the inflammatory activation and the exposure of the blood to a foreign material on top of the circuit dimension) and reducing drug clearance, by sequestration in membranes and circuits [14,71,72].

Table 1 provides recommendations for optimizing the loading dose in patients with increased Vd and maintenance dosing, guided by organ dysfunction, renal replacement therapy, and extracorporeal membrane oxygenation (ECMO).

Patients with chronic renal or hepatic dysfunction must be treated based on the same principles that were previously mentioned, regardless of pre-existing dysfunction: initial dosing according to the estimated level of drug Vd and maintenance therapy guided by organ dysfunction [13].

#### 2.2.1. Renal Failure

Acute kidney injury is a common sepsis-induced organ dysfunction that is associated with an increased risk of death and a longer length of hospital stay [73]. β-lactams are hydrophilic antibiotics with primarily renal elimination. The antibiotic prescription guidance in renal dysfunction is well established in chronic renal failure but is poorly validated in critically ill patients [74].

In a retrospective study of septic patients, Taccone et al. noted that, despite the use of an initial loading dose independent of organ dysfunction, most of the patients had suboptimal concentrations after their first dose of β-lactams, ranging between 16% (cefepime) and 75% (meropenem) [54]. To ensure initial antibiotic appropriateness, loading doses should be carefully planned during the first hours of therapy. Afterward, a dose adjustment (ideally adjusted to drug clearance) between the 2nd and 5th days of sepsis is usually required in most patients with acute kidney injury (with or without dialysis requirements) [75].

In septic patients with acute kidney injury, the evaluation of creatinine clearance is challenging due to variations in fluid balance, kidney function fluctuations, and creatinine metabolism [76] since serum creatinine and urinary output are imprecise markers of the glomerular filtration rate [77], which can lead to suboptimal antibiotic concentrations. To ensure proper exposure to time-dependent antibiotics and prevent toxicity, it is preferable to reduce the dosage rather than to decrease the dose frequency [46] (Table 1).

#### 2.2.2. Renal Replacement Therapy

The dosing of β-lactam antibiotics in patients supported with renal replacement therapy remains unclear. There is a wide range of renal replacement therapy characteristics that make the optimal antibiotic prescription difficult in the intensive care unit, such as the modality heterogeneity (from conventional intermittent hemodialysis to continuous renal replacement therapy or prolonged intermittent renal replacement therapy and from hemofiltration to hemodialysis or hemodiafiltration) and the prescribed filter types and settings (the intensity of renal replacement therapy, the use of predilution or postdilution fluid replacement, and effluent rates) [46,72].

In critically ill patients with acute kidney injury, the β-lactam doses defined for stable chronic renal failure patients may lead to underdosing [72]. On the other hand, the administration of conventional nonadjusted drug regimens has been suggested by some [49] to be associated, more than 50% of the time, with very high and toxic drug concentrations [78].

In the largest published pharmacokinetic study in critically ill patients receiving renal replacement therapy, a considerable variation in antibiotic dosing administration was noted [46]. No consistent association between the antibiotic concentration and dosage, the acute physiological disturbance, the estimated total renal clearance (defined as the sum of the total effluent rate and the patient’s intrinsic glomerular filtration rate), and the albumin concentration was observed, suggesting a role for TDM [79].

#### 2.2.3. Hepatic Failure

The effect of liver dysfunction on β-lactam concentrations is less well defined. Despite the liver’s strong impact on the elimination of lipophilic drugs, it may also influence the clearance of hydrophilic drugs. Patients with liver disease often have decreased renal clearance, even with normal serum creatinine. Thus, a dose reduction may be necessary, even for kidney-cleared antibiotics [80].

Hypoalbuminemia, resulting from the decreased hepatic synthesis and capillary leakage, contributes to an increase in Vd and the clearance of highly protein-bound antibiotics, namely ceftriaxone, ertapenem, and flucloxacillin [81]. These pharmacokinetic changes may result in a suboptimal drug concentration [81]. Again, in septic patients with liver dysfunction or hypoalbuminemia, the loading dose must be carefully planned to ensure adequate bacterial killing, and the maintenance dose should be guided by organ dysfunction/the main elimination pathways and possibly guided by TDM.

Data on PK during extracorporeal circulation to support severe hepatic failure are scarce.

#### 2.2.4. Extracorporeal Membrane Oxygenation

Antibiotic PK during ECMO support is affected by drug sequestration in the surface area of the tubing and membranes (again associated with an increase in the Vd), which is potentiated by the age of the circuit and reduced regional kidney and liver blood flows (reducing drug clearance) [14,82].

Shekar et al. compared the PK of meropenem in septic patients receiving or not receiving ECMO support. They found that the Vd of meropenem was not significantly higher in the ECMO group compared to the controls (0.45 ± 0.17 versus 0.41 ± 0.13 L/kg, *p* = 0.21) [83]. Consequently, those patients may need increased antibiotic loading doses. Moreover, a nonsignificant reduced clearance was noted (7.9 ± 5.9 versus 11.7 ± 6.5 L/h, *p* = 0.18), and this correlated with the creatinine clearance and the presence of renal replacement therapy, suggesting a role of creatinine clearance in guiding the maintenance dose [83]. A subsequent meta-analysis on this topic showed that modern ECMO circuitry has a minimal impact on the PK of hydrophilic antibiotics, leading the authors to conclude that PK changes in these patients mostly reflect the critical illness rather than the particularities of the ECMO therapy itself [84]. In opposition, in a large TDM study addressing 105 patients receiving continuous infusions of β-lactam antibiotics along with ECMO therapy, piperacillin and meropenem serum concentrations were significantly lower in ECMO patients (compared to non-ECMO patients), and almost half of patients treated with piperacillin did not reach the intended MIC targets [85]. The authors also found that higher doses of meropenem (6 g/d) or piperacillin (18 g/d) could overcome this insufficient dosage [85].

Still, data concerning PK and antibiotic dosing in ECMO are scarce and came from small single-center and neonate studies [86], making it difficult to guide the antibiotic dosage during ECMO therapy. The reliance on the conventional dosing of β-lactams is probably safe, and TDM may be useful while no robust PK data are available.

A recent study addressing meropenem PK included 14 patients on ECMO matched to 11 controls. The attainment of PK/PD targets (both 100% *f*T > MIC and 100% *f*T > 4 × MIC) was poor, but there was no difference between the ECMO and matched control patients [87].

### 2.3. β-Lactam-Related Adverse Events

**Figure 4:**
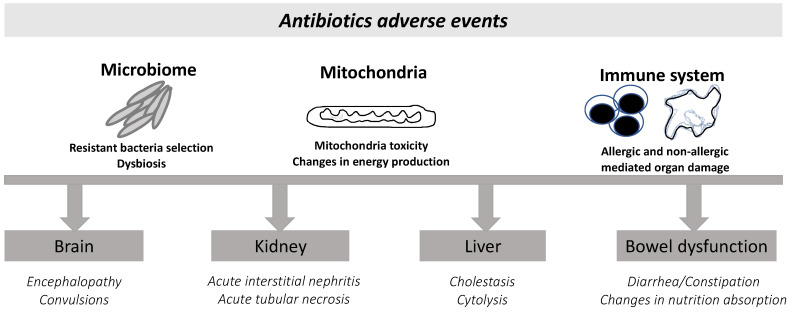
Antibiotic-related adverse events in patients.

Contrary to clinicians’ beliefs and the overall general belief, antibiotics are frequently associated with toxicity to patients (Figure 4) and not only toxicity to the community through resistance induction.

Controversial data coming from observational studies have associated unnecessary antimicrobial treatments with worse prognoses, suggesting that antibiotic administration does not have a neutral impact on human health, which is particularly relevant in the presence of unnecessary or prolonged antimicrobial treatments [88].

Overall, β-lactams are frequently considered safe, with negligible side effects, but data have been gathered that link this antibiotic class with potentially severe side effects, mostly dose-dependent [89] but also idiosyncratic, namely acute kidney injury, encephalopathy, and hepatic lesions [7] (Table 2). Direct organ-related adverse events, namely renal [90] and neurological [91] events have recently been reviewed by others.

We briefly discuss nephrotoxicity as well as neurological damage and focus on the adverse events associated with direct mitochondrial impacts, dysbiosis, and microbiome impairment. Overall, these toxicities are frequently ignored in conventional clinical practice or attributed to sepsis itself. However, they might be responsible for most of the adverse effects that are observed or even for persistent organ dysfunction in critically ill patients [88].

#### 2.3.1. Kidney Toxicity

Acute kidney injury is one of the most frequent adverse events associated with antibiotics. In everyday practice, vancomycin and aminoglycosides are considered the most direct culprits for de novo acute kidney injury, but β-lactams are also recognized as important nephrotoxins that can lead to acute tubular necrosis, acute glomerulonephritis, or acute interstitial nephritis. The latter is likely under-recognized, as the typical findings of fever, eosinophiluria, and rash have little sensitivity and specificity [93]. Contrary to other mechanisms of acute kidney injury, acute interstitial nephritis is harder to diagnose but relevant since, in selected cases, when persistent, patients might benefit from corticosteroid treatment [94].

Acute kidney injury due to acute interstitial nephritis is more frequent with some β-lactams such as piperacillin/tazobactam, although it has also been reported with amoxicillin [95]. This complication is most likely idiosyncratic, as it is not dose-dependent or related to prolonged infusions [96]. Interestingly, the combination of piperacillin with vancomycin appears to increase the risk of vancomycin nephrotoxicity, even after adjustment for potential confounders (8.1% for vancomycin versus 16.3% in the combination group, odds ratio: 2.48, *p* = 0.032). [97].

#### 2.3.2. Neurological Toxicity of β-Lactams

Even more frequent than acute kidney injury are encephalopathy and neurological toxicity [91]. There are three major presentations: epilepsy, confusion/delirium, and neuromuscular lesions. The increased risk of epilepsy appears to be related to the inhibitory activity of the GABA receptor, and therefore benzodiazepines and barbiturics are the drugs of choice. Epilepsy is mostly related to carbapenems, particularly imipenem; cephalosporins; and aminopenicillins. The spectrum of electrophysiological changes can also be very heterogeneous, ranging from myoclonus, asterixis, and seizures to nonconvulsive status epilepticus [15]. The mechanisms underlying the other neurological manifestations are not well understood but might be related to direct mitochondrial injury [98], as discussed below.

#### 2.3.3. The Impact of β-Lactams on Mitochondria

The mitochondrial toxicity of antibiotics has been known for a long time and might partially explain renal, ontological, and neurologic toxicity [99]. This effect, along with the immune paralysis observed after critical illness, is commonly regarded as secondary to sepsis or the infection process [100].

In 1990, using an animal model and in vitro studies, Tune et al. suggested that imipenem and a cephalosporin could induce, in a dose-dependent manner, irreversible changes in the kidney tubular function through the direct impairment of mitochondrial function [101]. More recently, also in an in vitro study, piperacillin was shown to directly induce neuron mitochondrial toxicity, with reductions in mitochondrial respiration, membrane potential, and ATP production [98].

The hepatic cytotoxicity of amoxicillin/clavulanate can also be partially attributed to mitochondrial dysfunction due to direct mitochondrial membrane breakdown, leading to the expulsion of cytochrome C, lipid peroxidation, and a decrease in the energy content [102], especially when used for a long period. Moreover, even the new β-lactams in development have been shown to have a direct effect on mitochondrial oxygen consumption, to inhibit complexes II and III of the mitochondrial electron transport chain, and to increase the production of reactive oxygen species, possibly contributing to hepatotoxicity [103].

#### 2.3.4. Microbiome Changes and Dysbiosis

Most of the data related to the effects of antibiotics on the microbiome and its association with disease status come from preclinical studies. For instance, animal studies have suggested a direct association between antibiotics, the loss of microbiome diversity, gut epithelial changes, and impaired mucosal IgA [104]. In addition, different antibiotics affect the microbiome, leading to different changes and the loss of diversity. For example, a combination that spares certain species such as Lactobacillus has been associated with a higher ability to control gut inflammation in a colitis model submitted to three different antibiotic regimens [105].

Data on the antibiotic disruptions of the human microbiome in different pathologies and antibiotic-induced dysbiosis are also being gathered. It is also important to recognize that the microbiome shapes not only global health but also the metabolism of drugs and the response to treatments [106]. In contrast, many antibiotic gene resistances are also harbored in the gut [107].

Separate parts of the human body harbor different microbiomes, including the skin, mouth, and lungs, which are all affected by antibiotics, although it is the microbiome of the gut (the most abundant and diverse) that is more prone to antibiotic-induced changes.

The effect of broad-spectrum antibiotics on the lung microbiome and its association with ventilator-induced injury were recently reviewed by Siwicki-Gieroba [108]. The same changes, pro-inflammatory modifications of the microbiome, were also shown to be associated with invasive mechanical ventilation itself [109].

One of the most frequent side effects of antibiotics is diarrhea. In a recent study involving 66 children with community-acquired pneumonia, the development of diarrhea was evaluated longitudinally and was associated with the dysbiosis of the microbiome [110]. This dysbiosis may also aggravate chemotherapy-related adverse events. A recently published animal study showed that antibiotic administration before chemotherapy was associated with an increased incidence of diarrhea and gut mucosa disturbances. This led to higher mortality after gut disruption with methotrexate. Interestingly, fecal transplantation improved the animals´ prognoses [111]. This again clarifies the role of the microbiome as an actor or contributor in the disease process.

### 2.4. Resistance

The use of antibiotics is commonly known to facilitate the emergence of resistance. It occurs both through the selection of resistant bacteria, usually in the gut [16], that can resist the antibiotic action (and multiply due to the decrease in competition) and through the induction of resistance mechanisms [112]. The microbial communities in the gut adapt continuously to the unpredictable changes that often occur. Accordingly, to survive in such dynamic habitats, microorganisms can switch the expression of different genes, leading to changes in their phenotypes within each population, which results in different subpopulations expressing different traits. In evolutionary biology, this phenotypic heterogeneity resulting from an unpredictably changing environment has been defined as bet hedging, a risk-spreading strategy [113]. Although this limits bacteria’s ability to predominate in a favorable environment, it may have a selective advantage upon a sudden environmental shift, such as an antibiotic insult.

Nevertheless, this proliferation of a specific phenotype that is able to resist the antibiotic insult comes with a metabolic cost. Resistance to antibiotics often implies changes in bacterial targets, that is, bacterial cell structures associated with the maximum efficacy to survive. These changes can impose a fitness cost on the bacterium when the antibiotic insult stops [114], which may limit their evolutionary success and may explain the late gut microbiome recovery [115] compared to the more adapted wild-type bacteria.

Accordingly, the evolutionary advantage of the mutation or plasmid expression associated with the repeated use of antibiotics may also be associated with an equal fitness cost, and the wild-type susceptible bacteria often will outcompete resistant strains in a different environment after antibiotic administration has ended or in the community.

However, repeated mutations during prolonged antibiotic exposure may lead to a compensatory evolution where a mutation combination contributes to the recovery of fitness by specific bacterial clones [116]. These will consequently be able to survive, even in the community, without antibiotics.

Resistant clones of bacteria such as Staphylococcus aureus, Klebsiella pneumoniae, Enterococcus faecium, Pseudomonas aeruginosa, and even Streptococcus pneumoniae can easily survive in the community, even without known antibiotic exposure.

#### Antibiotic Dosage to Suppress Resistance

The selection of antibiotic doses has been proposed as a strategy to prevent the emergence of resistance. This implies the consideration of the pharmacokinetic and pharmacodynamic properties and the possible use of antibiotic combinations [117]. In addition, avoiding antibiotics known to facilitate the emergence of resistance, for instance, quinolones to treat Pseudomonas aeruginosa, may help to limit this problem [117]. Some antibiotics within the same class may be more prone to facilitate the development of a resistant clone [118]. However, this remains controversial.

The suppression of resistance has been proposed to be related to higher antibiotic concentrations than those associated with efficacy. For β-lactams, the proposed best PK/PD index that decreases the emergence of resistance in Gram-negative bacteria is MIC ≥ 4 [119]. Still, this minimum exposure is probably largely related to each antibiotic per se, the length of exposure, immunocompetence, the bacteria being tested, the size of the inoculum, and the location of the infection. Moreover, differences in antibiotic PK (for instance, changing from intermittent to prolonged infusion) may influence the time–kill curves [120].

Theoretically, a higher antibiotic concentration should be able not only to suppress wild-type (sensible) bacteria but also mutants, with a higher MIC. This has been conceptualized in the mutant prevention concentration theory, which supports the use of drug concentrations above the lowest antibiotic concentration that prevents the replication of the least susceptible single-step mutant [121,122,123]. This concept is based on the idea that a large initial bacterial burden has a high probability of harboring a first-step mutant. Theoretically, the concentration window above the MIC but below the mutant prevention concentration would facilitate the proliferation of these mutant pathogens, facilitating the appearance of new mutations and the development of bacteria with two or more resistance mutations.

Although appealing, this mutant selection window hypothesis would depend on the ability to completely suppress the mutant clone to prevent it from proliferating and becoming dominant without competition. Although this complete suppression may seem appealing in vitro [124], it is probably not possible in vivo [125].

Consequently, achieving this higher target antibiotic concentration can reduce the total burden of bacteria, but it can also, paradoxically, facilitate the proliferation of the mutant single-step clone, which may lead to reduced antibiotic susceptibility. High doses of antibiotics foster opposing processes involving mutation-resistant strains and their release from the ecological competition [125]. Whether such an approach provides the best alternative for the control of the emergence of resistance depends on the relative strengths of these processes [125].

**Figure 5:**
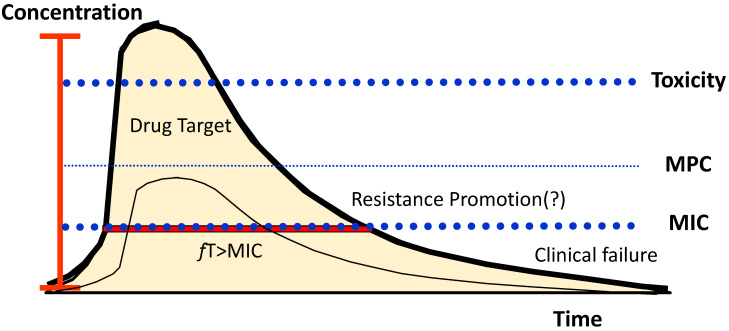
Concentration target of β-lactam antibiotics. Conceptually, the drug-free concentration should be above the MIC and the MPC for the necessary time to lead to bacteria death. A high concentration may lead to toxicity, and a low concentration may be associated with therapeutic failure. A concentration high enough to promote the death of the wild-type bacteria but below the MPC may promote the proliferation of mutants with lower sensibility, although this may depend on other factors (see the text for details). MPC—mutant prevention concentration; MIC—minimal inhibitory concentration; *f*T—free drug time above MIC. Adapted from [131].

An ideal antibiotic dosing regimen should, consequently, maximize bacterial cell killing while minimizing drug toxicity and the risk of resistance development throughout the antibiotic treatment duration (Figure 5). A dose modulation approach [125] should combine an initial high dose (the maximal tolerable dose [40]) to foster bacterial killing and improve efficacy with a low antibiotic dose (probably near the low margin of the therapeutic window) [125] to prevent toxicity and maintain ecological competition. At the same time, subtherapeutic concentrations should be avoided, and the duration of antibiotic therapy should be reduced to a minimum [126]. This combination strategy may be the best approach to decrease resistance development and maximize efficacy.

### 2.5. Challenges, Biosafety, and Clinical Translation

Overall, antimicrobial use in clinical practice has several caveats, and quality programs to solve them are challenging. Antimicrobial stewardship programs are now implemented in most modern hospitals, and they must consider not only the indication for a given antibiotic according to the patient infection and severity but also the unintended consequences of their usage in each setting, both for the patient and the environment. β-lactams’ unrestricted use has already led to the development of panresistant microbes, such as panresistant Pseudomonas aeruginosa and Klebsiella pneumonia. Outbreaks of such bugs in hospital settings, particularly in intensive care units and hematological or surgical wards, are especially cumbersome. In addition, there are already reports of panresistant microbes in community settings, which, together with hospital dissemination, present significant biosafety issues [127].

In the future, the challenge will be to develop new drugs against panresistant bacteria [128] with no or minimal environmental impacts, to find the best β-lactam for each β-lactamase inhibitor [129], and to consider the PK/PD, not only of the β-lactam but also of the β-lactamase inhibitor, as recently reviewed by Monogue et al. [130]. In addition, although these concepts are appealing, personalizing the antibiotic dose beyond organ-dysfunction-related adjustments is still not proven to be associated with better patient outcomes or even environment-centered outcomes. The design of randomized clinical trials to answer these questions is challenged, among other factors, by the need to include a significant number of participants and the requirement to ensure homogeneity between the patients and microbiological findings. Moreover, limited data exist addressing the cost-effectiveness of β-lactam TDM.

Overall, these challenges should not restrain the development of new drugs or their introduction into clinical practice, as recommended in recent years by the World Health Organization [128].

## 3. Conclusions and Future Directions

In recent years, β-lactam PK have largely been unveiled, and improved dosing is now possible. Unfortunately, dosing strategies to achieve specific therapeutic targets (measured by the PK/PD relationship) have produced conflicting results. Moreover, although the TDM of β-lactams remains attractive, the clinical benefits are still unproven. We believe that this is due to a lack of understanding of the real PK/PD targets. For TDM to be beneficial, there must be a clear relationship between the drug concentration and the intended effect. That is probably not the case with β-lactam antibiotics, as differences according to the infection itself, the bacteria, and the host may all lead to different therapeutic targets.

During the last few years, there has been a greater understanding of the potential adverse effects of β-lactams. These effects are not only related to an increase in bacterial resistance (environmental adverse events) but also to toxicity to the patients, organ injury, and ultrastructural cellular changes, especially in the mitochondria. A greater understanding of the role of the microbiome has contributed to understanding the collateral damage of antibiotics.

We believe that future studies should focus on the measurement of antibiotic PD, namely quantifying the death of bacteria as well as antibiotic concentrations and potential toxicity, using tests that allow rapid turnaround results for clinicians. This should also provide support for an even further reduction in antibiotic duration. Ideally, antibiotic therapy should be personalized to address the unique host–bacteria relationship and to overcome the bacteria’s virulence.

Studies on non-antibiotic approaches will likewise enlarge our therapeutic options. Replacing the microbiome and preventing the emergence of resistance should also be goals for therapy in the next few years.

## Figures and Tables

**Table 1:** Dose recommendations for β-lactams in critically ill patients with organ dysfunction.

Antibiotic	Loading Dose *	Maintenance Dose			
		Acute Kidney Injury	Continuous RRT	Hepatic Failure	ECMO
Flucloxacillin	2 g q4 h	1 g q4 h	1 g q4 h	2 g q4 h	ND
Ceftriaxone	1–2 g q12 h	1 g q12 h	1–2 g q12 h	1 g q12 h	1–2 g q12 h
Ceftazidime	2 g q8 h	1 g q8 h	1 g q8 h	2 g q8 h	ND
Cefepime	1–2 g q8–12 h	500 mg–1 g q12 h	1–2 g q12 h	1–2 g q8–12 h	ND
Piperacillin/tazobactam	4.5 g q4–6 h	4.5 q8 h or 2.25 g q6 h	4.5 g q8 h	4.5 g q6 h	4.5 q6 h
Ertapenem	1 g q12 h	500 mg q12 h	500 mg q8–12 h	1 g q12 h	ND
Imipenem	1 g q6 h	500 mg q6–8 h	500 mg–1 g q12 h	500 mg q6–8 h	750 mg–1 g q6 h
Meropenem	2 g q8 h	500 mg q12 h	500 mg q8 h	1 g q8 h	1–2 g q8 h
Ceftazidime/avibactam	2.5 g q8 h	1.25 g q8 h	1.25 g q8 h	2.5 g q8 h	ND

* In patients with evidence of an increased Vd, a high dose may be used for 48 h. For continuous infusion, consider the total daily dose. Select the higher dose in patients with severe infections and augmented renal clearance. Doses are based on the presumptive half-life (T_1/2_). Significant differences may occur. Vd—volume of distribution; RRT—renal replacement therapy; ECMO—extracorporeal membrane oxygenation; ND—no robust data. Adapted from reference [13].

**Table 2:** β-Lactam-related adverse events.

Side Effect	β-Lactam More Frequently Implicated	Dose-Related	Risk Factors	Note
Encephalopathy	Cefepime; Piperacillin/tazobactam	Yes	High risk for neurotoxicity if patient has acute kidney injury, previous neurological disease, hepatic encephalopathy, or advanced age	Higher risk with cefepime—up to 48% of exposed patients
Convulsions	Cefazolin; Cefepime; Imipenem	Yes		Compared to imipenem, lower risk for ertapenem, meropenem, and doripenem
Neuropsychiatric changes	Piperacillin/tazobactam	Yes		
Acute kidney injury	Piperacillin/tazobactam	Yes	Particularly in association with other nephrotoxins	Might relate to direct mitochondrial impairment
Acute interstitial nephritis	Piperacillin/tazobactam; Cephalosporins	No	Older age, increased incidence but reason is unknown	Non-IgE-mediated hypersensitivity reaction Can occur days to weeks after AB exposure Corticosteroids are recommended if resolution does not occur after stopping the culprit.
Acute kidney injury secondary to hemolytic anemia	Piperacillin/tazobactam; Ceftriaxone (However, all β-lactams have been associated with hemolytic anemia.)	Both	Previous hemolytic anemia associated with β-lactams	Very rare event (1 per million per year of incidence). High mortality
Renal obstruction due to crystallization	Amoxicillin; Ceftriaxone	Yes	Dehydration, acidic urine pH	Precipitation in renal tubules, lithiasis, and consequent obstruction
Hepatic lesion	AAC; Pip/taz	Yes	Other	Might be related to direct mitochondrial impairment
Dysbiosis	All	Yes	Critical illness	One of the most known consequences: clostridium difficile infection related to diarrhea associated with AB
Allergic reaction	All	Both	Unknown	Overestimation in the clinic led to a higher use of broad-spectrum antibiotics or second-line antibiotics (adapted from [92]).

## Data Availability

Not applicable.
